# Spermatogenesis after transplantation of adipose tissue-derived mesenchymal stem cells in busulfan-induced azoospermic hamster

**DOI:** 10.22038/IJBMS.2018.29040.7010

**Published:** 2018-07

**Authors:** Negar Karimaghai, Amin Tamadon, Farhad Rahmanifar, Davood Mehrabani, Alireza Raayat Jahromi, Shahrokh Zare, Zahra Khodabandeh, Iman Razeghian Jahromi, Omid Koohi-Hoseinabadi, Mehdi Dianatpour

**Affiliations:** 1 Stem Cells Technology Research Center, Shiraz University of Medical Sciences, Shiraz, Iran; 2 Department of Basic Sciences, School of Veterinary Medicine, Shiraz University, Shiraz, Iran; 3 Department of Clinical Sciences, School of Veterinary Medicine, Shiraz University, Shiraz, Iran; 4 Department of Medical Genetics, School of Medicine, Shiraz University of Medical Sciences, Shiraz, Iran; 5 Cardiovascular Research Center, Shiraz University of Medical Sciences, Shiraz, Iran; 6 Central Research Laboratory, Shiraz University of Medical Sciences, Shiraz, Iran

**Keywords:** Adipose tissue, Azoospermia, Cell therapy, Hamster, Mesenchymal stem cells

## Abstract

**Objective(s):**

Adipose tissue-derived mesenchymal stem cells (AT-MSCs) with more potent immunomodulatory effects, greater proliferative potential and secretion of growth factors and cytokines in comparison with bone marrow derived MSCs are more appropriate for cell therapy. The aims of the present study were to evaluate the histomorphometric effect of AT-MSCs allotransplantation on regeneration of germinal layer cells of seminiferous tubules in busulfan-induced azoospermic hamsters.

**Materials and Methods:**

In the present experimental case-control study, AT-MSCs were isolated from adipose tissue of two female and six male donor albino hamsters, and testes of the males were simultaneously used as negative control group. Six mature male recipient hamsters received two doses of busulfan with three weeks interval to stop endogenous spermatogenesis. Right testis of hamsters was intratubular injected with AT-MSCs via efferent duct 35 days after induction of azoospermia and was used as cell therapy group. The left testis without cell therapy was served as azoospermia group.

**Results:**

After 35 days, testes and epididymis in all groups were removed for histological evaluation. Histomorphometric analyses of AT-MSCs-treated testes and epididymis showed that the epithelial tissue of seminiferous tubules was normally repaired in most cell-treated seminiferous tubules, and spermatozoa were present in epididymis tubes in comparison with intact testes. The untreated seminiferous tubules and epididymis tubes of azoospermia group were empty.

**Conclusion:**

Allotransplanted AT-MSCs could successfully induce spermatogenesis in azoospermic seminiferous tubules of hamster. Therefore, AT-MSCs can be suggested as an attractive candidate in cell transplantation of azoospermia.

## Introduction

Cell therapy of azoospermia using germ cells and pluripotent and multipotent stem cells has been recently considered (1) as an important treatment modality of male infertility (2). Stem cell therapy has potential to be a choice for treatment of azoospermia and to repair inability and dysfunction of germ cells and also differentiate to or induce proliferation of germ cells (3). This technique attracted significant interest for treatment of sperm deformity and azoospermia.

Classically, stem cells are known as undifferentiated cells, with the ability to produce similar cells as themselves, and the ability of differentiation into a variety of specific somatic cells. Mesenchymal stem cells (MSCs) possess similar features including favorable proliferative capability, self-renewal, and differentiation potential. These cells are separated from bone marrow (4), adipose tissue (5-7), endometrium (8, 9), dental pulp (10), umbilical cord (11) and menstrual blood (12). The MSCs in bone marrow stromal comprise a restricted area, but it can be easily proliferated (13). These cells have the potential to proliferate and differentiate into other cells such as osteoblast (14, 15), chondroblast (16), adipocyte (17), and neuron-like cells (18, 19), which can be used for treatment of non-hematopoietic diseases.

*In vitro* studies showed that different kind of stem cells including MSCs can be differentiated into female germcell lineage (20). On the other hand, efforts in producing male germ cells from pluripotent cells *in vitro* were also successful (21). For instance, embryonic stem cells (ESCs) in *in vitro* conditions differentiated into Sertoli cells and primordial germ cells (22). Furthermore, germ line is derived from induced pluripotent stem (iPS) cells *in vitro* (23). Although these methods are developed for differentiation of pluripotent stem cells into male germ cells, but direct application of these cells in *in vivo* conditions has limitations including immunogenicity potential and ethical concerns of ESCs or risk of tendency to form teratoma in both EMCs and iPS cells. 

Therefore, application of MSCs for direct cell therapy of azoospermia can be selected as choice in future. In particular, the MSCs are shown to have the potential of differentiation into male germ cells *in vitro* (24). Although bone marrow MSCs (BM-MSCs) are used for the first time for *in vitro* and *in vivo* production of male germ cells (24), but some superior characteristics of adipose tissue-derived MSCs (AT-MSCs) gives them priority for cell therapy. Greater proliferative potential, more potent immunomodulatory effects and also greater secretion of cytokines and growth factors such as insulin like growth factor 1 (IGF-1), basic fibroblast growth factor (bFGF), and Interferon-gamma (IFN-γ) are the most important priorities of AT-MSCs in comparison with BM-MSCs for cell therapy (25).

On the other hand, cells with high division activities such as germ cells are susceptible to busulfan, a chemotherapeutic agent, which is applied for treatment of chronic myeloid leukaemia (26). It is shown that proliferation of spermatogonial stem cells of hamster can be disturbed by busulfan, and induction method of azoospermia is described in hamster (27). Furthermore, because of different anatomical position of efferent ducts on testis in hamster that exit directly from the apex (28), in comparison with rat and mice that exit the testis eccentrically (29), access to efferent ducts for intratubal injection of cells is easier. Therefore, hamster is selected as the model of azoospermia and this study was performed to evaluate the effect of AT-MSCs allotransplantation on induction of spermatogenesis in this model.

## Materials and Methods

***Animals***

**In the present experimental case-control study, two female and 12 male albino Syrian hamsters (** *Mesocricetus auratus*) weighing 110±8 g were obtained and housed in Center of Comparative and Experimental Medicine, Shiraz University of Medical Sciences under 12 hr light/dark cycle, and in a temperature and humidity-controlled room (22±1°C and 50±5%, respectively). The hamsters were fed with standard commercial chow diet *ad libitum*. 

This project was performed according to the instruction of experimental animal care of the Ethical Committee of Shiraz University and the project method had been reviewed and approved by the Committee of Vice Chancellor of Research, School of Veterinary Medicine, no: 1430. All efforts were made to minimize suffering during the experimental period.

***Isolation and culture of hamster AT-MSCs***

To isolate AT-MSCs, two male and two female hamsters were anesthetized by ether inhalation and then were euthanized by cervical dislocation. After shaving of abdominal and cervical skins and disinfection with 70% ethanol, incision was made on the skin, and the abdominal and cervical adipose tissues were completely removed. Samples from both sexes were mixed in the same falcon tube containing phosphate-buffered saline solution (PBS, Gibco) supplemented with 1% penicillin and streptomycin (Sigma). To remove the blood cells, the adipose tissues were washed with PBS solution again. The samples were sliced with a scalpel into small pieces (1 mm) and were incubated in 2 mg/ml collagenase type II (Gibco) solution at 37°C in water bath and were shacked every 5 min for 30 min. Then, the digested suspension was filtered and centrifuged for 5 min at 1500 rpm. The pelleted cells were re-suspended in 5 ml Dulbecco’s Modified Eagles Medium (DMEM, Gibco). The stromal vascular fraction suspensions were transferred into 25 ml flasks containing the cell culture media, DMEM, 10% fetal bovine serum (FBS, Sigma), and 1% penicillin and streptomycin. 

The flasks were incubated 48 hr in an incubator with 5% CO_2_ at 37 ^°^C and saturated humidity. After 48 hr, to remove non-adherent cells, the primary cell cultures were washed with PBS and the fresh DMEM supplemented medium was added. The culture medium was replaced every 72 hr. Using 0.25% trypsin (Gibco), the cell cultures in 80% confluency were subcultured into new flasks. 

The cells were sub-cultured two times to obtain a sufficient number for evaluation of stemness characteristics and cell therapy. Cells in the second passage were collected and counted using a hemocytometer. They were cryopreserved through the conventional method by dimethyl sulfoxide (DMSO; MP Bio, France) and were aliquoted into sterile cryovials at a density of 2×10^6^ viable cells/ml. The cryovials were gradually frozen respectively at -20°C for 60 min, -70°C for 24 hr, and finally in liquid nitrogen until the desired time.Before cell characterization or cell therapy, the frozen cryovials were quickly thawed in a 37°C water bath. Before the ice clump completely thawed, 1 ml of DMEM supplemented medium was added and centrifuged for 5 min at 1500 rpm. After re-suspension of the cells in fresh medium, they were cultured and subcultured just one time in the same condition and medium as explained above. Except cell morphology and plastic adherent characteristics of isolated cells, to confirm that the isolated cells were MSCs, the potential of differentiation to adipocytes and osteoblasts as well as their surface markers were detected by reverse transcription polymerase chain reaction (RT-PCR).

**Table 1:** Sequences of RT-PCR primers used to quantify the expression of adipose tissue-derived mesenchymal stem cells specific surface markers (CD29 and CD73) and hematopoietic stem cells specific surface markers (CD45) in hamster

Primer	Primer sequence	Amplicon length (bp)
CD29-F CD29-R	CAGTGAATAGCAACAATGAAGC ATAAGCAGCAAGGCAAGG	133
CD45-F CD45-R	TGATGCTATGCTGGAAGG GTATGAAGGAAGTCTCTGGAA	265
CD73-F CD73-R	TGGGAGGGAGAAGAGGATGGC GGAGAGTGGCAGGCAGAAATAAACC	336

***Adipogenic and osteogenic differentiations of AT-MSCs***

For adipogenic differentiation, DMEM, 15% FBS, 0.2 mM L-glutamine, 200 µM indomethacin, 100 µM L-ascorbic acid, and 100 nM dexamethasone, and for osteogenic differentiation, DMEM, 15% FBS, 10 mM glycerolphosphate, 200 µM L-ascorbic acid, and 100 nM dexamethasone were added separately into cell cultured plates with 80% confluency. Twice a week, the media were changed for 21 days. After three weeks, adipogenic and osteogenic differentiation were evaluated using Oil Red O and alizarin red staining, respectively. Briefly, for evaluating the generation of oil droplets in differentiated adipocytes, the cells were fixed in 10% formalin at room temperature for 10 min and washed twice with tap water. Next, Oil red O stain was added and incubated at room temperature for 1 hr. Finally, the cells were rinsed several times with tap water. In the flask of differentiated osteocytes, with 4% paraformaldehyde, the cells were fixed for 10 min. Then cells were incubated at room temperature for 20 min with 1% alizarin red and 1% ammonium hydroxide to bind calcium ions present in mineralized deposits. Then, wells were washed with 1 ml dH_2_O four times and 5 min each time; the water was replaced at each 5 min interval and air-dried (all reagents from Sigma).

**Figure 1:**
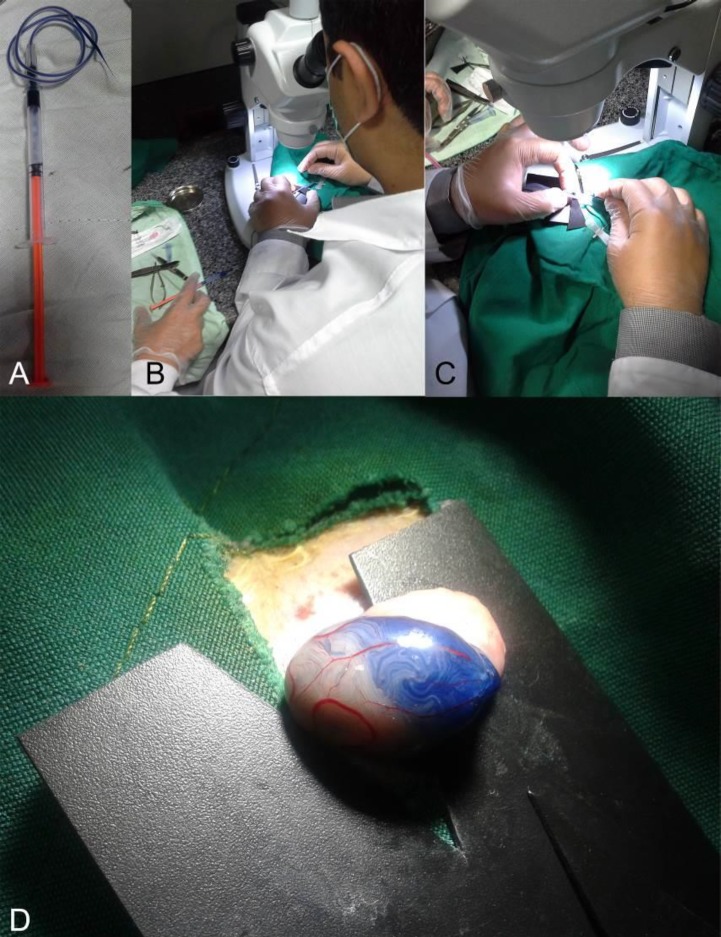
A, designed micro-injector consistent of a 1 ml syringe, tube of a 24 gage butterfly needle, and a pre-pulled glass pipette. It is filled with cell suspension that is stained with trypan blue. B, Injection was performed under a loop microscope by an expert operator with the help of assistant for pushing the syringe plunger gently. C, A triangular hard black plastic card was inserted underneath the efferent duct. D, Adipose tissue-derived mesenchymal stem cells (AT-MSCs) injected (10^6^ cells were mixed with trypan blue) into efferent duct of hamster testis (Seminiferous tubules were partially filled to demonstrate filling of the tubes)

***AT-MSCs surface markers***

RT-PCR was performed to evaluate the expression of AT-MSCs specific surface markers (CD29 and CD73) as well as the expression of hematopoietic stem cells specific surface marker (CD45) in the isolated cells based on the method previously described (15). Briefly, using the Column RNA Isolation Kit (Denazist-Asia, Iran), total RNA was extracted and its concentration was evaluated by spectrophotometer. Then, 12 thermal cycles including primer annealing for 30 sec at 20˚C, cDNA synthesis for 4 min at 42˚C, melting secondary structure and cDNA (complementary DNA) synthesis for 30 sec at 55˚C, and finally heat inactivation for 5 min at 95˚C were performed to synthesize cDNA of RNA samples by AccuPower Cycle Script RT PreMix Kit (Bioneer, Korea). After that, cDNA was mixed with forward and reverse primers of CD29, CD73, and CD45 (Table 1), PCR buffer, dNTPs, Taq DNA polymerase, MgCl_2_, and H_2_O and transferred to Thermocycler (Eppendorf, Hamburg, Germany). In 30 amplification cycles including denaturation at 95˚C for 30 sec, annealing at 64˚C for 30 sec and extension at 72˚C for 30 sec, RT-PCR was run and continued with primary denaturation for 2 min at 95˚C and final extension for 5 min at 72˚C. Presence of considered bands of PCR products was visualized in gel electrophoresis and by DNA safe stain under UV radiation by gel documentation system (UVtec, Cambridge, UK).

***Induction of azoospermia***

Five male hamsters were used for induction of azoospermia and two doses of 10 mg/kg busulfan (Busilvex®, Pierre Fabre Medicament, Boulogne, France) were IP injected with 21 days interval. According to the previously described method of azoospermia induction in hamster (27), the model was induced 35 days after the last busulfan injection. The remained five male hamsters served as negative control.

***AT-MSCs transplantation***

AT-MSCs transplantation into seminiferous of right testis of azoospermic hamsters as cell therapy group was performed according to our previously explained techniques (15). Briefly, simultaneous to AT-MSCs trypsinization, detachment and mixing with sterile trypan blue (1:1, v/v), the azoospermia-induced hamsters were anesthetized by IP injection of ketamine (100 mg/kg, Woerden, Netherlands) and xylazine (7 mg/kg, Alfazyne, Woerden, Netherlands). In dorsal recumbency, their abdominal area was surgically disinfected by povidone iodine scrub, and 1 cm midline abdominal incision was made to achieve the peritoneum and then testis. Suspension of AT-MSCs was loaded into the injection set including a polyethylene tube connected to a 1 ml syringe, and a pulled pipette was attached into the tube (Figure 1A). After fixation of testis, using a stereomicroscope (Model SZN, Optika, Italy) (Figure 1B), the tip of pulled pipette was carefully inserted into the efferent duct (Figure 1C) and 10^6^ cells in 100 μl suspension gently injected and observed by gradual flowing the blue colors into seminiferous tubules (Figure 1D). The testis was returned to the abdominal cavity. The abdominal wall and skin were closed by sutures. The untreated left testes were used as azoospermia group.

**Figure 2:**
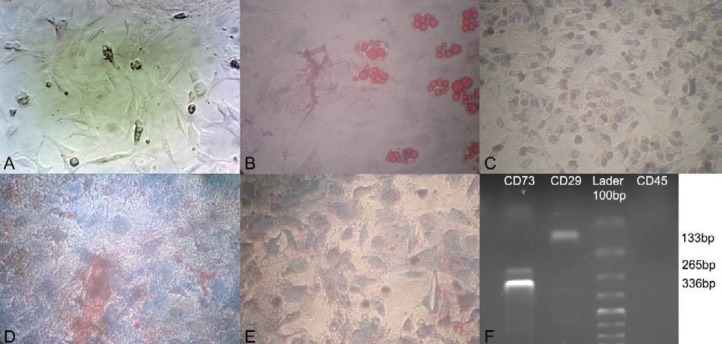
Characterization of hamster adipose tissue-derived mesenchymal stem cells (AT-MSCs). A, Attached large spindle-shaped, fibroblast-like morphology in passage 3, which confirmed the morphology of the isolated MSCs (×200). B, AT-MSCs of hamster cultivated in osteogenic medium and stained with alizarin red (×200) and C, its control without osteogenic media (×200). D, AT-MSCs of hamster cultivated in adipogenic medium that were stained with Oil Red O at day 21 after induction (×200) and E, its control without adipogenic media (×100). F, mRNA expression of specific mesenchymal cell surface marker (CD29 and CD73) in AT-MSCs of hamster compared with non-expression of specific hematopoietic marker (CD45)

***Histomorphometric analysis of testes***

The hamsters were euthanized with ether and cervical dislocation 35 days after cell transplantation. The epididymis tails and testes were collected and fixed in a 10% formalin buffer solution. After fixation, samples were dehydrated and embedded in paraffin, and five vertical sections with 5 µm thickness were made from each block. The slices were rehydrated and stained with hematoxylin-eosin. Using light microscope, spermatogenic activity was examined in testis and epididymis sections of cell therapy and azoospermic groups. Then, using the histomorphometric method as previously explained (27), in 10 circular transverse sections of seminiferous tubules from a different region of the testes of all three groups, histomorphometric indices were compared. Inner, outer and total diameters were measured. Furthermore, calculated areas of the cellular and luminal regions and cross sectional area of the tubules, and spermatogenic index based on the presence and number of spermatogenic cells was evaluated.

***Statistical analysis***

After evaluation of normal distribution of the data of histomorphometric indices of seminiferous tubules by Kolmogorov-Smirnov test, means and standard error (SE) of the data were analyzed by one-way ANOVA and Tukey *post hoc* test (SPSS for Windows, version 11.5, SPSS Inc, Chicago, Illinois). By Mann-Whitney U test, the spermatogenesis index of seminiferous tubules was compared between groups. *P*≤0.05 was considered to be statistically significant.

## Results

***Characterization of AT-MSCs***

Spindle-shaped fibroblast-like morphology of isolated and cultured cells from the hamster adipose tissue as the first criteria and plastic adherence of cells to the culture flasks as the second criteria primarily showed that the isolated cells were AT-MSCs. AT-MSCs proliferation started 3-4 days after incubation until reaching an 80% confluence (Figure 2A). For further confirmation of the MSCs characteristics in the isolated cells, the adipogenic and osteogenic differentiation capacity of AT-MSCs were evaluated. After culture of AT-MSCs in adipogenic and osteogenic differentiation media separately, the cells showed the presence of intracellular lipid droplets of adipocytes and differentiated toward osteoblasts as respectively verified by positive staining with Oil Red O (Figures 2B and C) and alizarin red staining (Figures 2D and E). To approve the expression of surface marker of MSCs of hamster on isolated AT-MSCs, the cells were analyzed using RT-PCR. Figure 2F displays positive expression for MSCs marker (CD29 and CD73) and negative expression for hematopoietic stem cell marker (CD45).

***Histological assessment of spermatogenesis***

**Figure 3:**
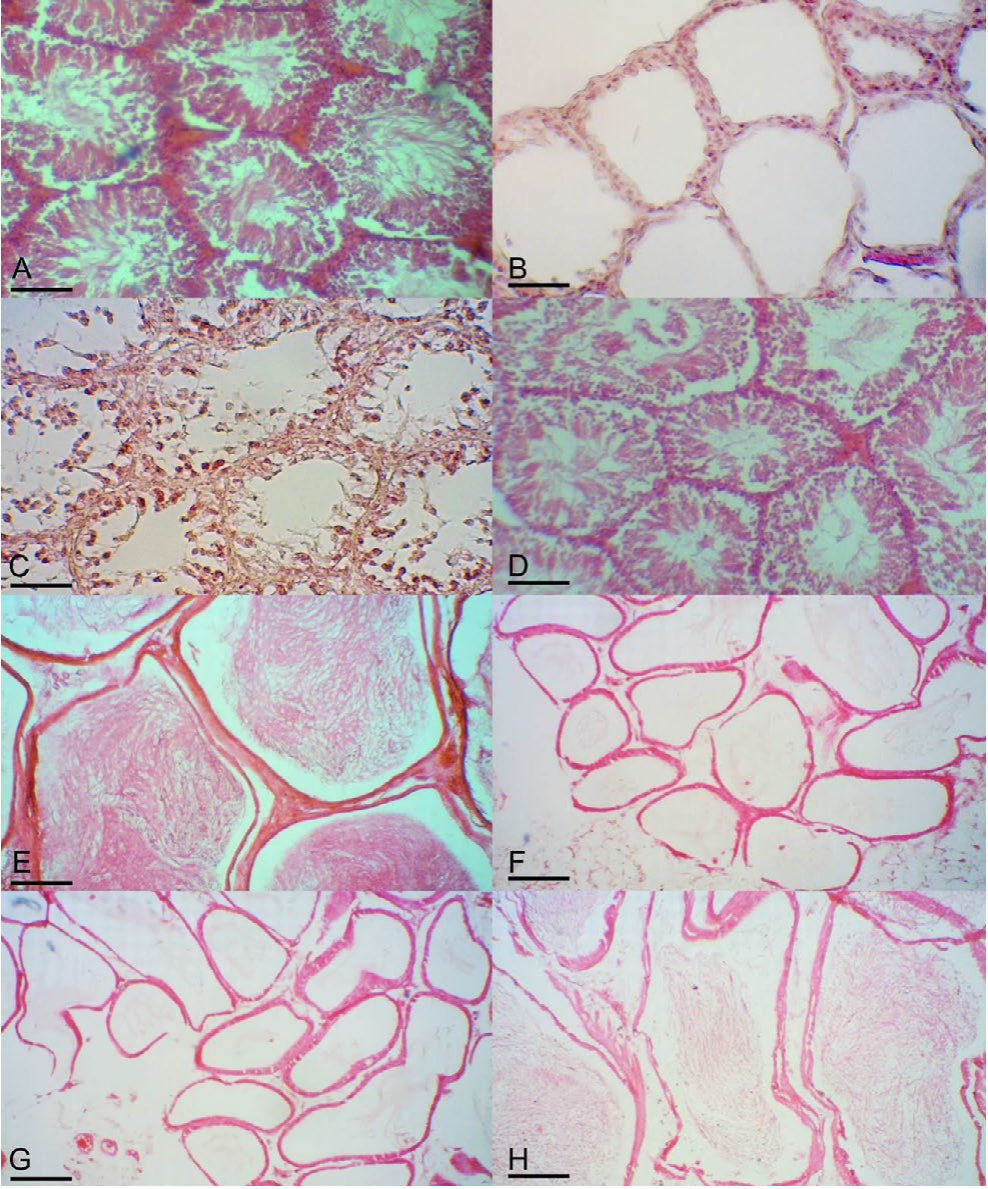
A, Seminiferous tubules of normal control hamster with condensed spermatogenic epithelium. B, Seminiferous tubules of busulfan-treated azoospermic control were partially empty and without germinal layer cells indicating the absence of spermatogenesis. C, Seminiferous tubules of treated hamster with adipose tissue-derived mesenchymal stem cells (AT-MSCs). Some of tubules appeared starting spermatogenesis but not completely repaired. D, In the other seminiferous tubules of treated hamster with AT-MSCs, presence of different kind of germinal epithelial cells was observed and tubes were filled up with spermatogenic cells. Sections of epididymis tubules of E, normal control, F and G, busulfan-treated azoospermic control, and H, AT-MSCs treated hamsters. Hematoxylin and eosin staining and all indices are 50 μm

Normal intact hamsters had condensed germinal epithelium in their seminiferous tubules (Figure 3A). While, after treatment with double injection of 10 mg/kg busulfan, the seminiferous tubules of the testes in azoospermia group after 35 days, which were not treated with AT-MSCs, were empty and their spermatogenesis process was completely disrupted and revealed degenerative changes such as germinal epitheliums degenerations and seminiferous tubular atrophy in all tubules (Figure 3B).

**Figure 4:**
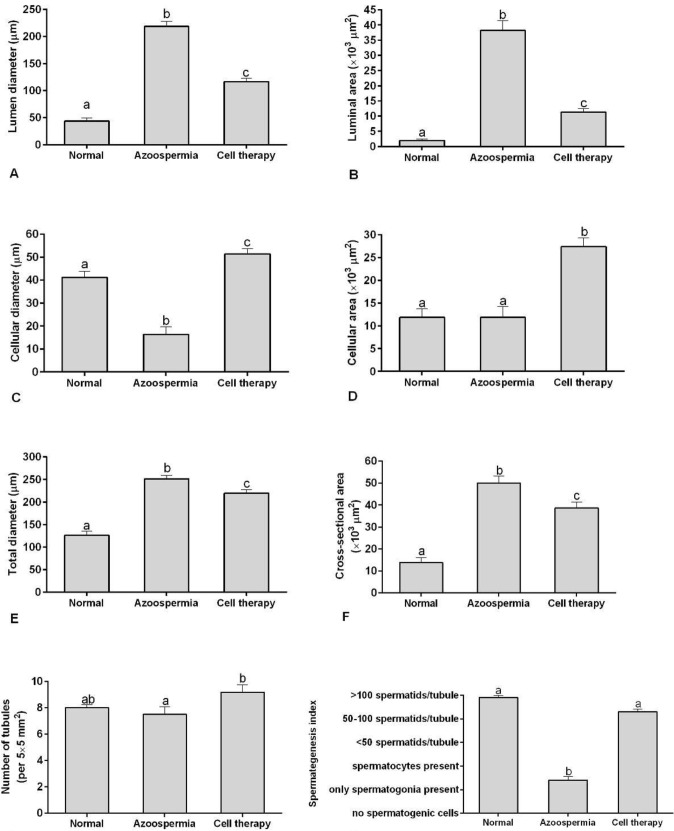
Mean and standard error of histomorphometric indices of seminiferous tubules in busulfan-induced azoospermic testis treated with adipose tissue-derived mesenchymal stem cells (AT-MSCs) (cell therapy) in comparison with busulfan-treated testes (azoospermia) and intact normal testis (normal) in hamster. A, lumen diameter (μm), B, luminal area (μm^2^), C, cellular diameter (μm), D, cellular area (μm^2^), E, total diameters (μm), F, cross sectional area of the tubule (μm^2^), G, number of tubules per 5×5 mm^2^, H, spermatogenesis index of seminiferous tubules. a, b, c different superscript letters show significant differences between groups (*P*<0.05)

On the other hand, in some treated seminiferous tubules of azoospermic hamster with AT-MSCs, Sertoli cells and the cells of lower layers of germinal epithelium were formed but no spermatid and spermatozoa were observed in seminiferous tubules (Figure 3C). In addition, the presence of spermatogonia in some seminiferous tubules with AT-MSCs transplantation was observed. Moreover, the other tubules in the AT-MSCs therapy group appeared to be filled up with germinal cells including spermatogonia, primary spermatocytes, spermatids and sperms (Figure 3D). 

In normal intact group, all of epididymis tubes were filled by spermatozoa (Figure 3E).

However, in the epididymis of busulfan-treated azoospermic group, no spermatozoa were observed after 35 days (Figure 3F). Also, in this group thickening of the walls of some epididymis tubes was observed (Figure 3G). In contrast, most of the epididymis tubes of AT-MSCs-treated group had spermatozoa, but with lower condensation of spermatozoa than the normal group (Figure 3H).

***Histomorphometric assessment of spermatogenesis***

Histomorphometric measurements of sperm- atogenesis indices in seminiferous tubules indicated that the lumen diameter and luminal area of the seminiferous tubules in hamsters with AT-MSCs transplantation were less than the azoospermic hamsters (*P*=0.001 and *P*=0.001, respectively) and more than normal hamster (*P*=0.001 and *P*=0.001, respectively; Figures 4A and 4B).

In addition, cellular diameter and cellular area of the germinal layers of seminiferous tubules in hamsters with AT-MSCs transplantation were more than azoospermic and normal control groups (*P*=0.001 and *P*=0.001, respectively; Figures 4C and 4D). However, cellular diameter of normal control group was more than azoospermic hamsters (*P*=0.001), but cellular area of these two groups were not different (*P*>0.05). Moreover, total diameter and cross sectional area of the seminiferous tubules in hamsters with AT-MSCs transplantation were less than the azoospermic hamsters (*P*=0.001 and *P*=0.001, respectively; Figures 4E and 4F) and also the measured values in both groups were more than the normal control (*P*<0.05). On the other hand, mean and SE of the number of tubules per 5×5 mm^2^ in AT-MSCs therapy group was more than azoospermia (*P*<0.05; Figure 4G); but, this index was not different between these groups and control intact group (*P*>0.05). Furthermore, spermatogenesis index of seminiferous tubules in hamsters with AT-MSCs transplantation was not different from normal control hamsters (*P*>0.05; Figure 4H), but this index in these groups was more than that in the azoospermic hamsters (*P*=0.001 and *P*=0.001, respectively).

## Discussion

In this study, the effects of AT-MSCs allotransplantation in the testis of busulfan-induced azoospermic hamsters were evaluated. Intratubal injection of AT-MSCs induced spermatogenesis in azoospermic seminiferous tubules. Interestingly after 35 days, spermatogenesis index of seminiferous tubules in hamsters with AT-MSCs transplantation was not different from normal intact hamsters. Consistent with our findings, in the same model of azoospermia in hamster, BM-MSCs allotransplantation induced spermatogenesis in most cell-treated seminiferous tubules (15). Furthermore, several *in vivo* studies have been performed to evaluate the spermatogenesis induction potential of MSCs in rat and mice animal models. In a group of these studies, BM-MSCs have been used for induction of spermatogenesis. In mice model, there are controversies in the findings of BM-MSCs transplantation in azoospermic mice, for instance it is reported that BM-MSCs could not differentiate into sperm (30), but in other studies, transplanted mouse BM-MSCs have been used to generate germ cells *in vivo* (23, 31). On the other hand, in rat model of azoospermia, BM-MSCs allotransplantation enhanced endogenous fertility recovery in both busulfan-induced and testicular torsion model of azoospermia induction and also by either inter- or intra-tubal injection of the cells (16, 32-35). The next group used AT-MSCs for induction of spermatogenesis. Consistent with our findings in hamster model, intra-tubal injection of AT-MSCs in rat model of busulfan-treated azoospermia led to recovery of fertility (5, 36). In the last group of studies, spermatogenesis was induced using xenotransplantation of human umbilical cord MSCs in seminiferous tubule of immunodeficient mice (37) or combination of *in vitro* differentiation of induced pluripotent stem cells from mice and humans into germ cells and also their transplantation was performed to obtain advanced differentiated spermatozoa (38). Therefore, although BM-MSCs is a common source for cell therapy, but our success in treatment of azoospermia using AT-MSCs in hamster along with the previous reports in rat model of azoospermia demonstrate the potential of this source for treatment of human azoospermia.

Although it is not confirmed in this study if AT-MSCs differentiate to spermatozoa or not, but if they do not have this ability, AT-MSCs transplantation may induce reconstitution of the tubular microenvironment in azoospermic hamster, which helps remained inactivated germinal cells to proliferate in the host seminiferous tubules. Seminiferous tubules provide dynamic and cyclic regulation of spermatogenesis in which the role of Sertoli cells is significant. Recently it was shown that in *in vitro* co-culture system, Sertoli cell could mediate differentiation of male germ cell-like cells, which were derived from human umbilical cord MSCs (39). In addition, Sertoli cells are immune tolerant cells (40) and they can cause survival and protection of the allotransplanted donor AT-MSCs against immune or inflammatory reaction. On the other hand, hypoimmunogenic characteristic of MSCs makes them suitable for allogeneic transplantation (41). Furthermore, MSCs produce immunosurveillance or immunosuppression upon transplantation (42). Interestingly, related to treatment of azoospermia, IV allogeneic BM-MSCs transfusion immune-modulated antisperm antibody production after testis rupture in mice (43), which shows the other therapeutic potential of MSCs in infertility treatment. Therefore, although the possible mechanisms of healing of azoospermic hamster by AT-MSCs are unclear, three mechanisms might be responsible to recover testicular function during the tissue regeneration process by AT-MSCs. The first possibility is that AT-MSCs may differentiate into the spermatozoa via appropriate induction conditions as it has been shown in rat (36). The next mechanism that is not yet confirmed is that AT-MSCs secrete growth factors to stimulate the inactivated spermatogonia stem cells or Sertoli cells to restore the spermatogenesis. And the final suggested mechanism is that AT-MSCs merged with the endogenous spermatogonia stem cells recover the spermatogenesis, which also need to be studied.

Furthermore, histomorphometric analysis of the treated seminiferous tubules showed the other point needs to be considered in cell therapy of azoospermia. Interestingly, the number of tubes per unit area decreased in azoospermic group that was caused by increase of the area of the seminiferous tubules before transplantation. This disorder maybe created because of decreased cellular layers, which caused the decrease of the tubal structure and collapsing of some tubules under intratubular hydrostatic pressure of hamster seminiferous tubules. Increase of the spaces in testis may result in increase of the diameter of the other tubes and also decrease in intratubular hydrostatic pressure. It is necessary to express that although the role of this pressure in the mechanism of spermatogenesis has not been clarified, but it can be one reason for increase of the diameter of cellular layer in AT-MSCs treated tubes. As a point, histomorphometric analysis, which has not been mentioned by the previous studies, is increase of the number of tubules per unit area in AT-MSCs treated tubes. This alteration caused by the busulfan therapy may lead to complications and decrease of the volumes of intertubular spaces. Furthermore, increase of total area of tubes during azoospermia induction may reduce the ability of contraction of contractile myofibroblast cells in peritubular layer, which along with decrease of intratubular hydrostatic pressure, probably resulted in observed decrease in concentrations of spermatozoa in epididymis of AT-MSCs treated hamsters. Interestingly, cellular diameter of normal control group was more than azoospermic hamsters, but cellular area of those two groups were not different. Mathematical description of this event is the increase of area of seminiferous tubules in the azoospermic hamsters; moreover, greater space for non- affected Sertoli cells with busulfan in germinal layer made the cells to cover more two-dimensional space than the normal group.

## Conclusion

AT-MSCs therapy could induce regeneration of damaged germinal layers in seminiferous tubules of azoospermic hamsters. However, our histomorphometric findings showed that AT-MSCs therapy could not completely reconstruct the normal structure of seminiferous tubules after busulfan induction of azoospermia in hamster. The present findings may increase the possibility of using AT-MSCs for treatment of azoospermia in human.

## Conflicts of Interest

The authors have no conflict of interest.
